# Osteogenic Activity and Bone Matrix Mineralization Induced by *Vitis vinifera* Leaves Extract in Human Osteoblastic Cells

**DOI:** 10.1002/fsn3.70785

**Published:** 2025-08-17

**Authors:** Maria Giovanna Rizzo, Marika Cordaro, Dario Morganti, Enrico Gugliandolo, Roberta Fusco, Ylenia Marino, Gianluca A. Franco, Salvatore Cuzzocrea, Rosanna Di Paola, Sabrina Conoci

**Affiliations:** ^1^ Department of Chemical, Biological, Pharmaceutical and Environmental Sciences University of Messina Messina Italy; ^2^ Department of Biomedical and Dental Sciences and Morphofunctional Imaging University of Messina Messina Italy; ^3^ CNR‐IMM Institute for Microelectronics and Microsystems Messina Italy; ^4^ Department of Veterinary Sciences University of Messina Messina Italy; ^5^ Department of Chemistry “Giacomo Ciamician” University of Bologna Bologna Italy

**Keywords:** bone tissue regeneration, osteogenic pathway, *Vitis vinifera* leaves extract

## Abstract

The agricultural production of *Vitis vinifera* leaves represents the majority of agricultural remains among the least studied and valorized in the wine industry. *Vitis vinifera* contains a variety of bioactive compounds, for example, polyphenols with positive effects on human health. Grape polyphenols can modulate the expression of specific bone matrix proteins, promoting osteoblast differentiation and bone mineralization. In this study, we investigated the effect of *Vitis vinifera* leaves extracts (VVLE) on bone matrix formation in the human fetal osteoblast cell line (hFOB 1.19). Specifically, we assessed: (i) *cytotoxicity* of VVLE using the MTT assay; (ii) mineralization quantification by staining calcium deposits with Alizarin Red S (ARS) and the enzyme activity by using the alkaline phosphatase (ALP) assay; and (iii) the osteogenic expression levels of specific markers in the processes of bone mineralization (BMP‐2, ERK, ALP, β‐GLAP, SPP1, and BSP) by quantitative real‐time PCR (qRT‐PCR) and Western blot. The results showed that the addition of VVLE in the medium stimulated the calcium deposits and the expression of genes involved in bone formation through the induction of *ERK*‐mediated *BMP2* expression. These data suggest that *VVLE* may positively influence physiological responses underlying bone tissue regeneration.

## Graphical abstract

*Vitis vinifera* leaves extract stimulates the proliferation, calcium deposits formation, and the expression of genes involved in bone matrix formation in human osteoblast cells. *Vitis vinifera* leaves extract, containing mixed bioactive compounds, may have combined and potential effects on bone tissue regeneration.

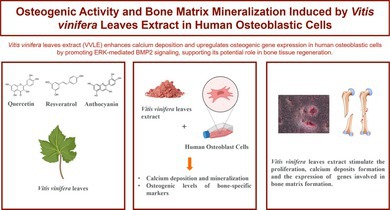

## 1 Introduction

Plant extracts are studied for their bioactive components, which support physiological functions in the human body (Samtiya et al. 2021). Plant extracts from fruits, seeds, or leaves exhibit significant health‐promoting effects; in fact, they are excellent antioxidants, anti‐inflammatory, anticancer, and can have a systemic protective effect (Majeed and e Bhat 2022; Mancuso et al. 2020). Grape ( *Vitis vinifera* ) and grape product consumption have been linked to a number of health benefits, including cardiovascular health, cancer prevention, and neurocognitive protection (Grace Nirmala et al. 2018). Grapes contain an amount of polyphenols such as quercetin, resveratrol, and anthocyanins (Anderson et al. 2011; Ariffin et al. 2022).

Bioactive compounds, especially polyphenols such as resveratrol and quercetin, have been shown to enhance bone morphogenetic protein 2 (BMP‐2) expression and stimulate the (Extracellular Regulated Kinase) (ERK) pathway, promoting bone mineralization and remodeling. BMP‐2, a key regulator of bone metabolism, activates small mother against decapentaplegic (SMAD)‐dependent pathways, including ERK (Extracellular signal‐regulated kinase) and the expression of transcription factors such as Runx2 and Osterix. These transcription factors are essential for osteoblast differentiation and bone matrix protein synthesis (Trzeciakiewicz et al. 2009).

Several proteins play a role in the processes of cell differentiation and activation and mineralization (Sommer et al. 1996); their expression is strongly regulated. Osteopontin and bone sialoprotein have been identified as the primary sources for mineralization and are necessary for the start of the process. In particular, bone sialoprotein acts as a crystal nucleator, while osteopontin may be related to ensuring that the right type of crystal forms. Osteocalcin and osteonectin are not present in the areas of major crystal formation, but in the totally mineralized matrix. Osteocalcin plays a role as a chemoattractant for osteoclasts, while osteopontin and bone sialoprotein can promote the binding of osteoclasts thanks to the presence of an Arg‐Gly‐Asp (RGD) motif (Roach 1994).

Grapevine derivative products have received great attention, leading to their valorization in food industries (Nzekoue et al. 2022). The agricultural production of *Vitis vinifera* leaves represents the majority of agricultural waste and the least studied and valorized waste in the wine industry. They are added to the soil as organic fertilizer, but could be investigated for their use as a natural resource for human health (Balli et al. 2021; Ramadan et al. 2017). Recent studies have documented that *Vitis vinifera* leaves, often considered waste, are rich in polyphenols and antioxidants, with comparable or even higher levels than other agricultural by‐products such as grape pomace or grain husks (Nzekoue et al. 2022). This makes vine leaves an underexplored yet valuable source of bioactive compounds with potential health benefits beyond their current low valorization in the wine industry.

This study aimed to assess the potential benefits of VVLE on bone health by evaluating the physiological responses of the human fetal osteoblast cell line (hFOB), focusing on matrix formation and bone‐related gene expression over time.

## 2 Results

### 2.1 Cytotoxicity

**FIGURE 1:**
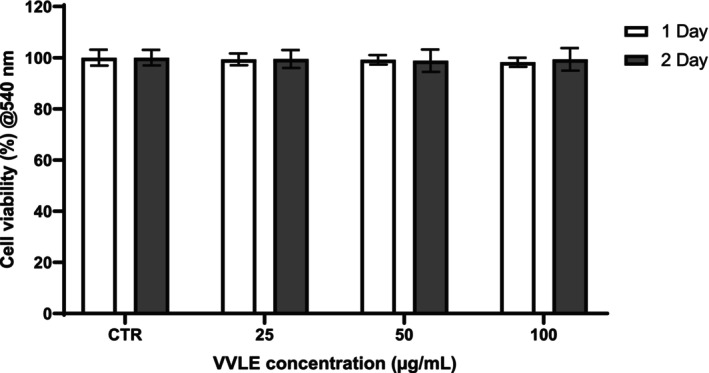
Cytotoxicity cell viability expressed in % compared to the control (CTRL, only cells) after 1 and 2 days. The data were derived from three independent experiments conducted in triplicate. Each data point represents the mean of three replications ± standard deviation (SD).

Cytotoxicity of VVLE in hFOB cells was assessed using MTT assays, with cells cultured in the presence of different concentrations (25–100 μg/mL). Cells grown without VVLE were used as a control. The results showed that at all concentrations tested and up to 2 days, the percentage of viability was equivalent to the control (Figure 1), indicating the biocompatibility of the compound.

**FIGURE 2:**
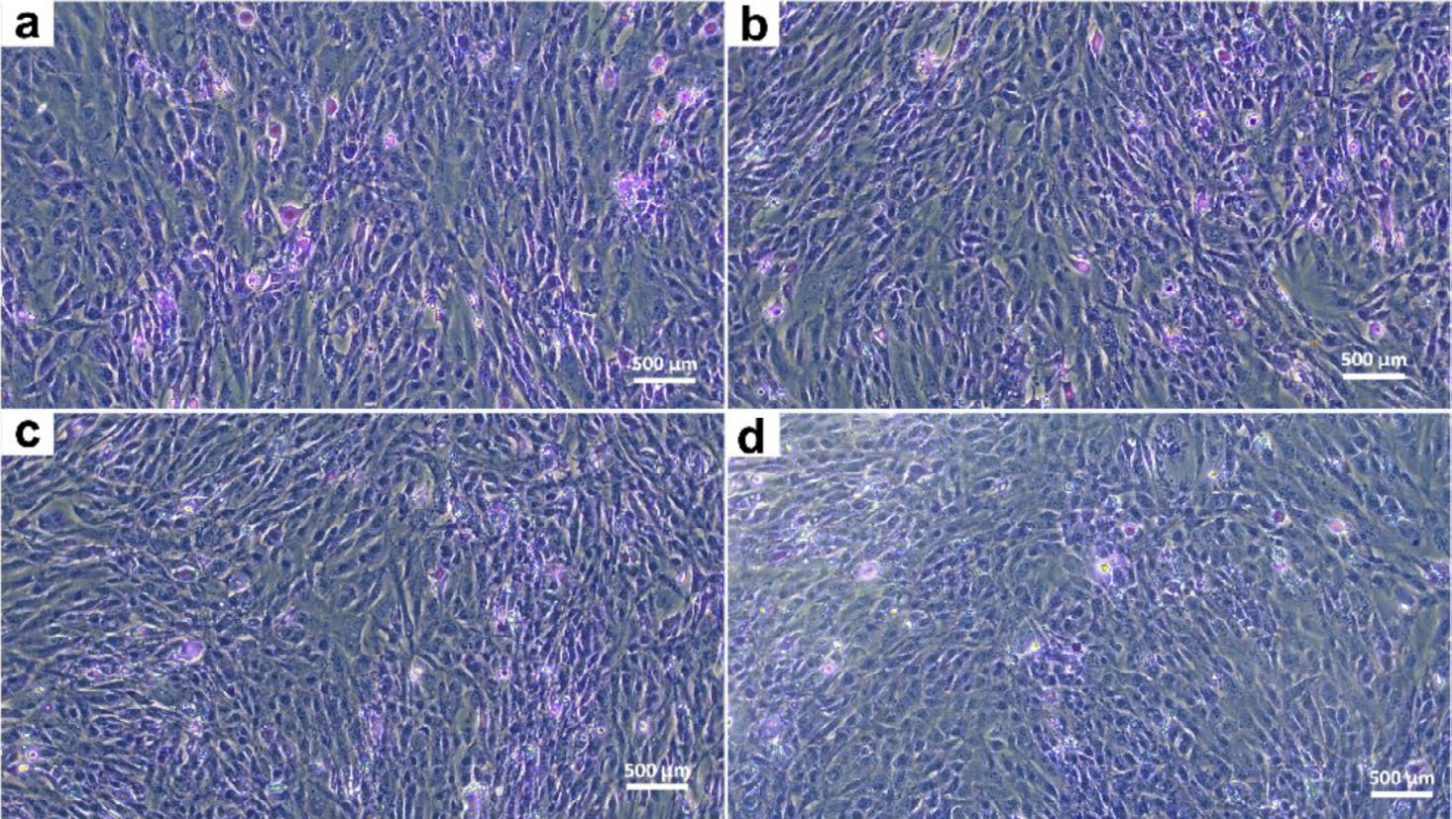
Optical microscopy of hFOB cultured in VVLE by crystal violet staining. (a) CTR‐only cells; (b) 25 μg/mL VVLE; (c) 50 μg/mL VVLE; (d) 100 μg/mL VVLE. Scale bar: 500 μm. The data were derived from three independent experiments conducted in triplicate.

To confirm the confluency distribution and viability compared to the control, cells were stained with crystal violet and images acquired at high magnification (Figure 2a–d).

### 2.2 Extracellular Matrix Mineralization

**FIGURE 3:**
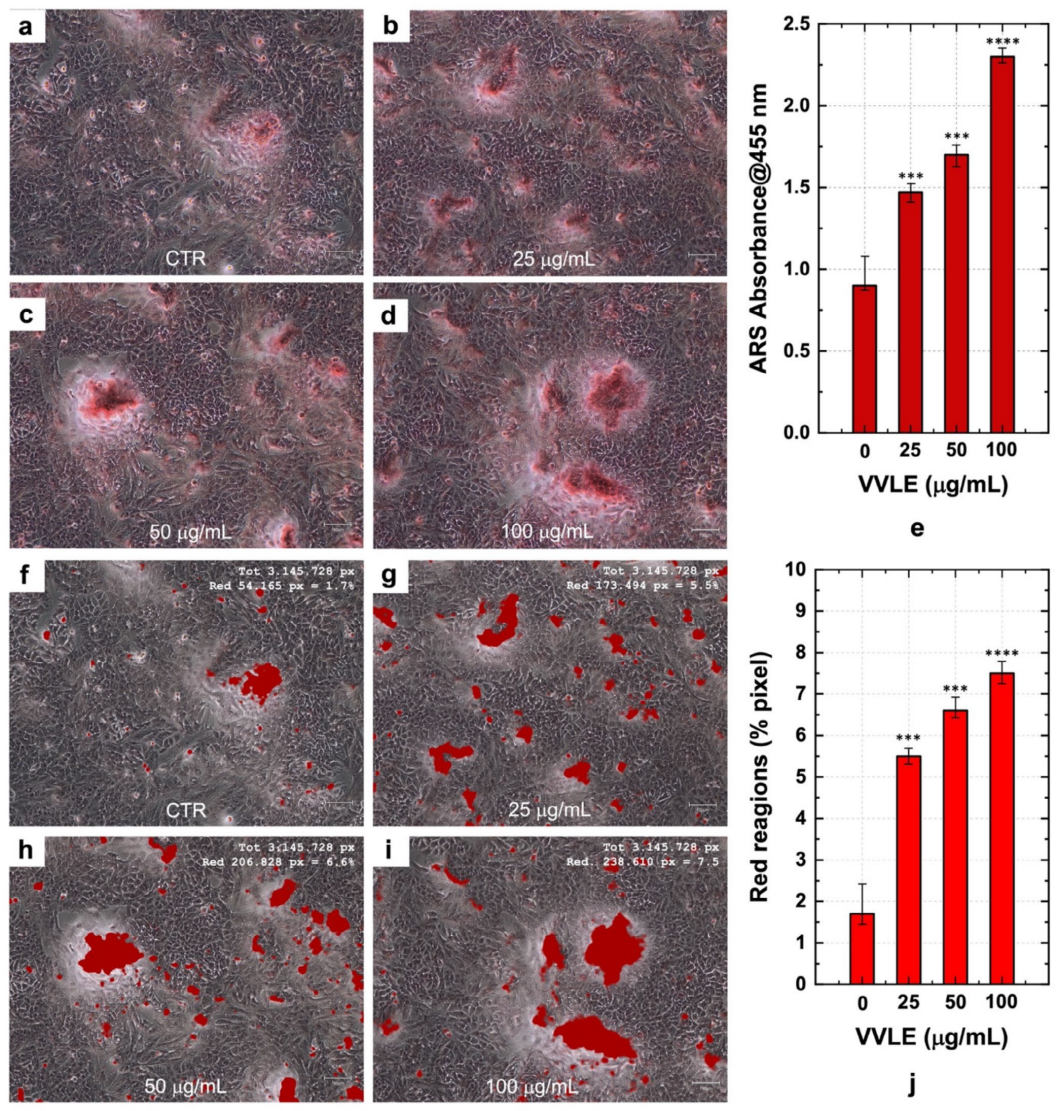
Extracellular matrix mineralization (a, b) and calcium deposits quantification (f, j). Alizarin Red S staining of matrix mineralization of cells grown without VVLE—CTRL (a); with 25 μg/mL (b), 50 μg/mL (c), 100 μg/mL (d), VVLE (e); ARS quantification in absorbance 405 nm. Scale bar: 100 μm. The images showed (f–i) marking of calcium amount present in some portions of the images in a–d (scale bar: 100 μm), and in (j) the intensity of marked areas in percentage of marked pixels as a function of VVLE concentration. All the images were derived from three independent experiments conducted in triplicate; the statistical analysis was reported as ***p < 0.001, ****p < 0.0001.

The effect of VVLE on the bone mineralization process was analyzed by observing the increase in calcium deposits by ARS staining (Figure 3a–e). The images reported in Figure 3a–d are representative of three independent experiments where a calcium accumulation deposition is shown in a monolayer of hFOB cells. The ARS dye highlighted the affinity to calcium ions and was used as an indicator of mineralization in the cells' monolayer.

Cells cultured without osteogenic factors and in a medium without VVLE were used as a control. These showed an initial calcium deposition that represented the normal mineralization process of osteoblastic cells in the absence of VVLE. While cells maintained in a medium without osteogenic factors but only in the presence of VVLE at a concentration of 25, 50, and 100 μg/mL showed a dose‐dependent calcium accumulation. The results indicated that after 14 days of incubation, the first formations of calcium deposits were visible in the control. A greater accumulation of deposits was visualized in cells incubated in the presence of VVLE. In particular, calcium‐rich deposits were larger with increasing concentration of VVLE. Quantitative analysis of ARS staining, reported in Figure 3e, confirmed the dose‐dependent effect of VVLE on mineralization. The absorbance values measured at 450 nm significantly increase with VVLE concentration, reaching the highest value at 100 μg/mL. This result supports the visual observation and demonstrates that VVLE enhances mineralized matrix formation in a concentration‐dependent manner.

Calcium deposits were numerically quantified by calculating the percentage of red pixels present in the image (Figure 3f–j). The dimensions of the analyzed images are the same for all samples, in order to obtain images having the same number of total pixels. The images showed a highlighted area of 1.7% in the control, 5.5% using 25 μg/mL VVLE, 6.6% using 50 μg/mL VVLE, and an area of 7.5% using VVLE 100 μg/mL. In Figure 3j, the concentration of calcium deposits (expressed as % of the red pixels) as a function of the VVLE concentration is reported. The values show an increasing trend in the range of the analyzed concentrations. The control sample was associated with a VVLE concentration equal to 0. The standard deviation values indicate a clear increase in calcium deposits above the experimental error, compared to control cells without VVLE (Figure 3j).

The dose‐dependent increase in calcium deposits observed with VVLE suggests a stimulatory effect on mineralization, as demonstrated by intensified Alizarin Red S staining and quantitative image analysis.

**FIGURE 4:**
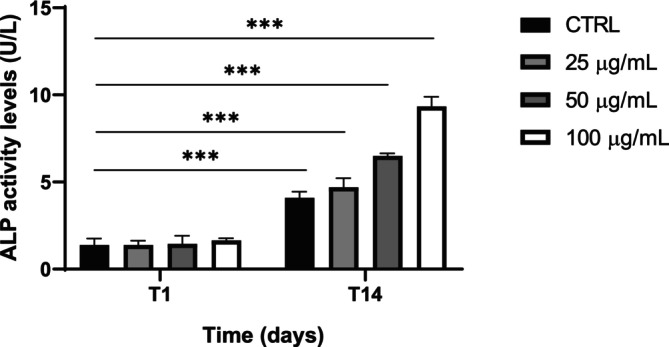
Alkaline phosphatase (ALP) enzymatic activity. ALP level measured at Day 1 (T1) and Day 14 (T14) in cells incubated with VVLE compared to the control. The images and data are representative of three independent experiments conducted in triplicate. The statistical analysis was reported as ***p < 0.001. The data were derived from three independent experiments conducted in triplicate.

To complement the analysis of the extracellular matrix formation, ALP enzymatic activity was assessed at Day 1 (T1) and Day 14 (T14) in the presence of VVLE at concentrations of 25, 50, and 100 μg/mL (Figure 4). A significant increase in ALP activity was observed at T14, particularly at the highest concentration (100 μg/mL). Statistical comparisons at Day 14 confirmed that ALP levels were significantly higher in all samples incubated in the presence of VVLE compared to the control group (*p* < 0.001). These results indicated that VVLE enhances enzymatic activity related to the mineralization process in a dose‐ and time‐dependent manner.

### 2.3 Gene Expression Profiles

The expression of genes related to various aspects of bone differentiation and mineralization was analyzed, including: (i) early differentiation marked by alkaline phosphatase (ALP), (ii) bone mineralization and calcium deposition through bone matrix proteins such as osteopontin (SPP1) and bone sialoprotein (BSP), (iii) regulation of bone formation and resorption by osteocalcin (β‐GLAP), and (iv) signaling pathways critical for osteogenesis; in particular, bone morphogenetic protein‐2 (BMP2) and Extracellular signal‐regulated kinase (ERK) (Mizuno and Kuboki 2001; Silvent et al. 2013; Trzeciakiewicz et al. 2009).

BMP‐2 expression was significantly upregulated at all concentrations (25, 50, and 100 μg/mL) starting from Day 7, following a clear dose‐dependent trend. Specifically, expression levels were 2.8 ± 0.4 (25 μg/mL), 3.2 ± 0.3 (50 μg/mL), and 4.8 ± 0.4 (100 μg/mL) compared to the control (1.0 ± 0.29). At Day 14, this pattern persisted, with values increasing to 3.9 ± 0.4, 4.5 ± 0.2, and 6.0 ± 0.2, respectively, indicating a sustained and progressive stimulation over time.

ERK gene expression exhibited a similar profile. At Day 7, significant upregulation was observed at 50 μg/mL (1.72 ± 0.15) and 100 μg/mL (2.15 ± 0.19); while a more moderate increase was detected at 25 μg/mL (1.08 ± 0.23). By Day 14, ERK expression further increased to 3.44 ± 0.20 (50 μg/mL) and 6.17 ± 0.26 (100 μg/mL), with 25 μg/mL reaching 2.26 ± 0.24, confirming a robust, dose‐dependent activation of this pathway.

ALPL, an early marker of osteoblastic differentiation, was the only gene significantly upregulated as early as Day 1, showing a marked increase at 100 μg/mL (1.31 ± 0.06) compared to control (1.00 ± 0.09). This early activation suggests that bioactive compounds within VVLE may promote early osteoblastic commitment by enhancing enzymatic activity and increasing the availability of inorganic phosphate required for mineral deposition. This effect might be mediated by signaling pathways such as BMP‐2/Smad, which are known to regulate ALP expression in osteoblastic cells (Liang et al. 2012). At Day 7, ALPL expression remained elevated at 50 μg/mL (0.98 ± 0.04) and 100 μg/mL (1.12 ± 0.13), and by Day 14, a dose‐dependent increase was evident across all concentrations: 1.08 ± 0.10 (25 μg/mL), 1.36 ± 0.11 (50 μg/mL), and 1.31 ± 0.06 (100 μg/mL). While lower concentrations elicited a response comparable to control, higher doses promoted earlier and more sustained activation.

β‐GLAP expression did not show significant changes at either Day 1 or 7 across all concentrations. At Day 14, a slight but statistically significant upregulation was observed at 50 μg/mL (1.42 ± 0.17) and 100 μg/mL (1.41 ± 0.08); whereas expression at 25 μg/mL (1.08 ± 0.23) remained comparable to control, indicating that this late‐stage marker is responsive only to higher doses and prolonged exposure.

SPP1 expression was not affected at Day 1. At Day 7, SPP1 expression showed a moderate increase at 25 μg/mL (2.00 ± 0.23), although not statistically significant, while significant upregulation was observed at 50 μg/mL (2.44 ± 0.29, *p* < 0.01) and 100 μg/mL (3.60 ± 0.30, *p* < 0.001). At Day 14, this dose‐dependent trend continued, with expression levels rising to 2.76 ± 0.23, 3.80 ± 0.38, and 4.50 ± 0.24, respectively. These findings suggest that VVLE exerts a strong and sustained stimulatory effect on osteopontin expression, especially at higher concentrations.

**FIGURE 5:**
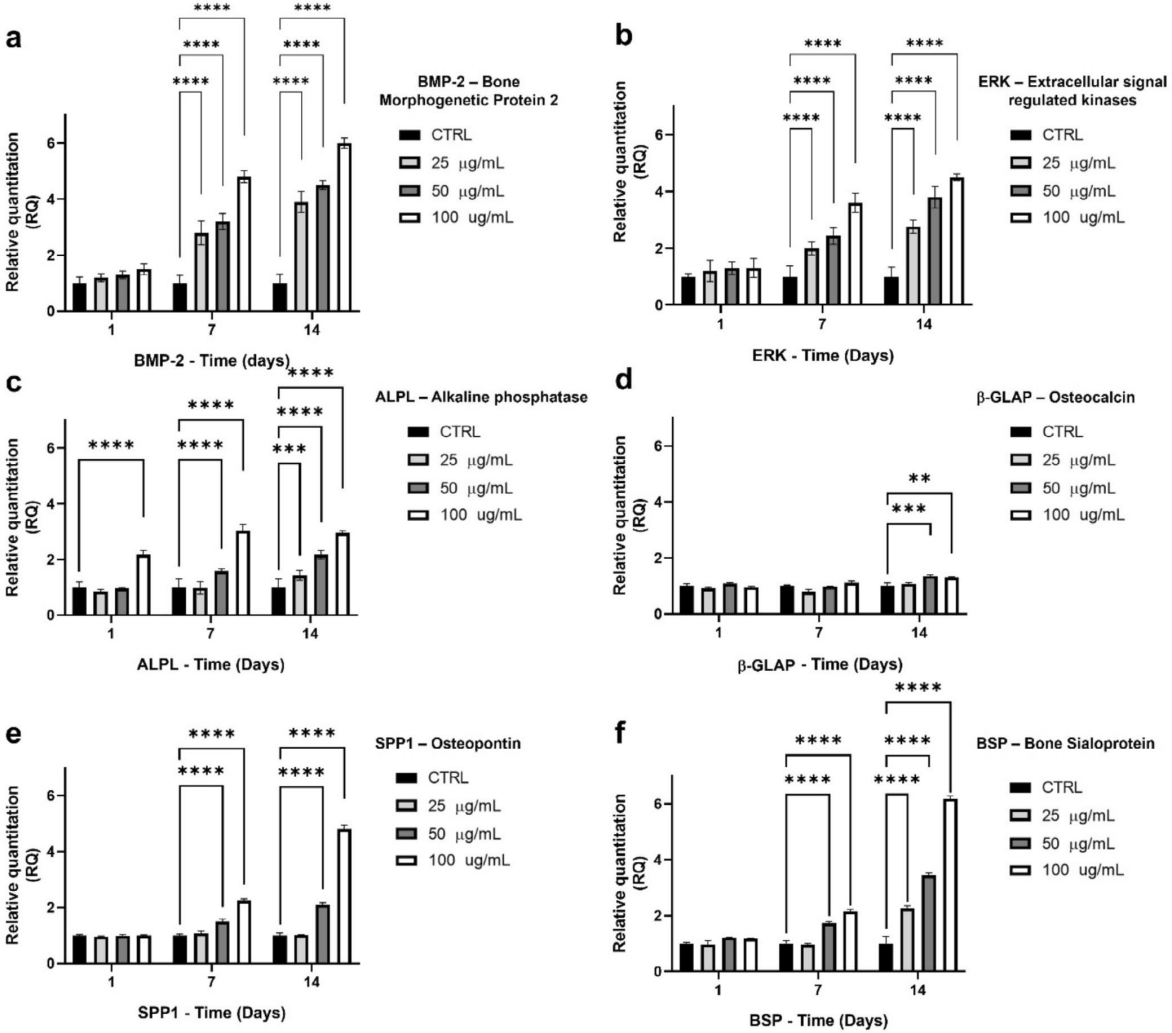
Gene expression profile of hFOB. Data expressed as relative quantitation (RQ) and glyceraldehyde 3‐phosphate dehydrogenase (GAPDH) was used as a control for normalization. The gene expression was monitored at the early (1 day), middle (7 days), and late (14 days) time points. (a) BMP‐2, (b) ERK, (c) ALP expression, (d) β‐GLAP, (e) SPP1, and (f) BSP expression in hFOB cells cultivated in VVLE at 25, 50, and 100 μg/mL. The statistical analysis was reported as **p < 0.01, ***p < 0.001, ****p < 0.0001. The data were derived from three independent experiments conducted in triplicate.

BSP expression increased moderately at Day 7 at 50 μg/mL (1.57 ± 0.08) and more markedly at 100 μg/mL (3.03 ± 0.22), while expression at 25 μg/mL (0.98 ± 0.11) was not significantly different from the control. By Day 14, all concentrations induced a significant upregulation: 1.42 ± 0.18 (25 μg/mL), 2.17 ± 0.15 (50 μg/mL), and 2.95 ± 0.08 (100 μg/mL). The time‐dependent emergence of a stimulatory effect even at the lowest dose suggests that BSP may be particularly responsive to prolonged exposure and plays a critical role in the late stages of matrix mineralization (Figure 5).

### 2.4 Protein Expression Profiles

As we did for gene expression, we evaluated the same parameters for proteins involved in bone differentiation and mineralization.

**FIGURE 6:**
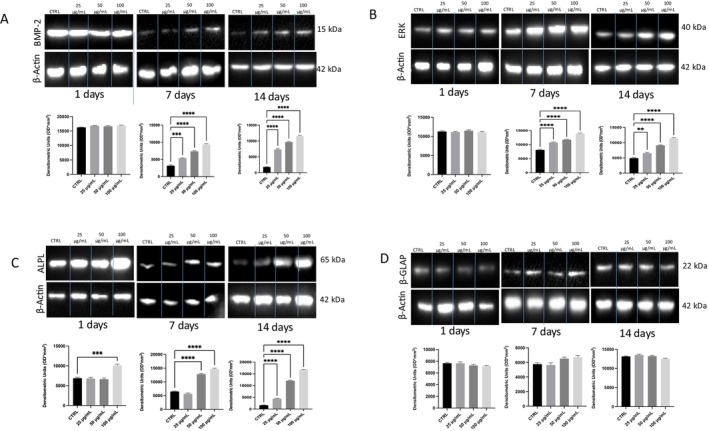
Protein expression in hFOB after VVLE treatment. Western blot analysis was used to evaluate (a) BMP‐2, (b) ERK, (c) ALPL, and (d) β‐GLAP expression in hFOB cells after VVLE treatment from 1 to 14 days at different concentrations. The statistical analysis was reported as **p < 0.01, ***p < 0.001, ****p < 0.0001.

As shown in Figure 6a for BMP‐2 and Figure 6b for ERK, expression was similar at all concentrations after 1 day. However, expressions were significantly increased compared to the control at all concentrations on both Days 7 and 14 in a dose‐dependent manner. As shown in Figure 6c, ALPL protein expression was significantly increased at concentrations as early as 1 day and up to 14 days. The intermediate concentration had a positive effect starting from 7 days and up to 14 days. However, the lowest concentration was significant only at 14 days.

In Figure 6d, β‐GLAP protein expression showed a significantly increased expression compared to the control only at the highest concentration at 14 days.

**FIGURE 7:**
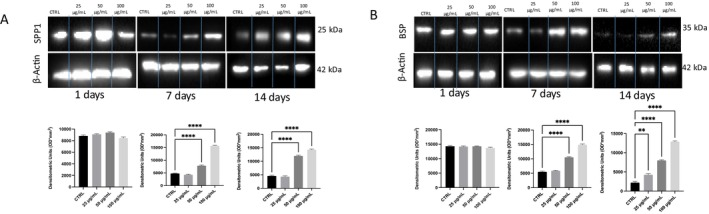
Protein expression in hFOB after VVLE treatment. Western blot analysis was used to evaluate (a) SPP1 and (b) BSP expression in hFOB cells after VVLE treatment from 1 to 14 days at different concentrations. The statistical analysis was reported as **p < 0.01; ****p < 0.0001.

SPP1 protein expression, in Figure 7a, showed a significant increase compared to the control when hFOB were treated with VVLE at the highest concentration at both 7 and 14 days.

hFOB treated with VVLE did not show any difference in BSP in Figure 7b expression; at Day 1, on the other hand, we appreciated a significant increase after 7 days at both 50 and 100 μg/mL, and a more significant increase at 14 days at all concentrations.

## 3 Discussion

Plant extracts, fruits, seeds, and leaves can also be used to maintain bone health (Jolly et al. 2018). The leaves of *Vitis vinifera* represent the least studied and valorized waste from vine cultivation and the wine industry (Nzekoue et al. 2022). However, the bioactive compounds present in vine leaves, such as carotenoids, tocopherols, polyphenols, and phytosterols, showed a bone‐forming effect on the expression of bone‐specific matrix proteins (Valentino et al. 2021). In this study, we investigated the effects of VVLE on bone regeneration using the human fetal osteoblast cell line in terms of bone matrix formation and the expression of bone‐related genes over time.

In this study, we used human fetal osteoblastic cells (hFOB 1.19) due to their robust osteogenic potential, genetic stability, and ability to maintain differentiation capacity across multiple passages, minimizing interdonor variability and improving consistency in osteogenic differentiation studies. Unlike primary osteoblasts, which may exhibit donor‐to‐donor heterogeneity and limited expansion potential, hFOB cells provide a standardized and reproducible system to assess the osteogenic effects of natural compounds such as VVLE (Marozin et al. 2021; Morganti et al. 2024; Oliveira Pinho et al. 2021).

However, other models can better mimic the natural bone microenvironment. Bone marrow‐derived mesenchymal stem cells (BM‐MSCs), for example, have multilineage differentiation potential and can differentiate into osteoblasts, offering a physiologically relevant model (Mazzoni et al. 2021). Similarly, mouse preosteoblast (MC3T3‐E1), widely used in bone research, follows a well‐characterized differentiation process but is of murine origin, limiting its translational relevance. While BM‐MSCs provide a valuable system for studying bone remodeling, their differentiation potential can be influenced by donor variability and culture conditions. hFOB cells, on the other hand, offer a standardized and well‐characterized model, making them particularly suitable for controlled preliminary studies on osteogenic responses.

The cytotoxicity of hFOB cells, evaluated by MTT assay, did not indicate a toxic effect using VVLE, avoiding any cellular morphological changes at all concentrations tested, both at 1 and 2 days. Similarly, Coelho MC et al., using osteoblastic MC3T3‐E1 cells, described that the seed extract of grape did not cause cell morphological changes but rather increased cell adhesion (Coelho et al. 2023).

The dose‐dependent increase in calcium deposition observed by Alizarin Red S staining indicated that VVLE would have a stimulatory effect on extracellular matrix mineralization. This finding was further confirmed by the significant increase in alkaline phosphatase (ALP) activity, an essential marker for the early stages of osteoblastic differentiation. ALP plays a crucial role in promoting mineralization by releasing inorganic phosphate, which in turn combines with calcium ions to form hydroxyapatite, the major mineral component of bone. The increase in ALP activity observed at both the enzymatic and gene expression levels suggests that VVLE could activate specific osteogenic pathways by facilitating phosphate deposition within the bone matrix.

Bioactive compounds such as polyphenols and flavonoids have shown potential to enhance osteogenesis by modulating cellular activities and signaling pathways that contribute to bone health. Polyphenols, known for their antioxidant and anti‐inflammatory properties, can stimulate the expression of specific bone matrix proteins, thereby enhancing mineral deposition and osteoblastic activity. Recent studies (Bernar et al. 2023; Li et al. 2023; Qureshi et al. 2014; Rizzo et al. 2017; Rizzo et al. 2023) have shown that polyphenol‐functionalized scaffolds can significantly enhance osteogenic differentiation, aligning with our findings and suggesting that polyphenolic compounds in VVLE play a critical role in promoting bone matrix formation and mineralization.

Safari et al. demonstrated that a scaffold with phosphorylated polycaprolactone enhances osteogenesis in AD‐MSC by activating ALP and ERK1/2 signaling, highlighting the role of phosphate groups in mineralization (Safari, Aghazadeh, et al. 2022).

In another study, it was shown that the treatment of osteoblasts with controlled concentrations of inorganic phosphate increased BMP‐2 expression through activation of the ERK1/2 signaling pathway. This approach highlighted the key role of ERK1/2 in promoting osteoblastic differentiation and extracellular matrix mineralization (Tada et al. 2011).

In line with previous studies demonstrating the crucial role of ERK1/2 signaling and BMP‐2 expression in osteoblastic differentiation and extracellular matrix mineralization, our results showed a similar trend with an increase in the expression of osteogenic genes such as BMP2, ERK, ALP, SPP1, and BSP following VVLE treatment. This suggests that *Vitis vinifera* leaf extracts may modulate the same signaling pathways, contributing to bone matrix formation.

In particular, BMP2 expression increased in a dose‐ and time‐dependent manner, confirming its centrality in promoting mineralization and osteoblastic differentiation. This finding is consistent with Safari et al., where phosphates and bioactive components activated BMP‐2 through ERK1/2, underlining the close correlation between these molecular pathways.

The ERK pathway is known to activate transcription factors such as Runx2, which are essential for the expression of bone matrix proteins such as SPP1 and BSP, both significantly overexpressed in our study.

Finally, our work confirms an overexpression of ALP, an essential marker of the early stages of osteoblastic differentiation, strictly associated with phosphate deposition in the bone matrix. This aligns with observations from previous studies, which highlight the role of phosphate groups in the activation of mineralization, even in models based on functionalized scaffolds (Calabrese et al. 2023).

These results highlight the importance of VVLE as a valuable natural extract with osteogenic properties, particularly due to its ability to modulate mineralization and osteoblast differentiation through key physiological pathways. The VVLE may provide an innovative approach to support bone health and address degenerative bone conditions. The results presented here lay the foundation for the future development of VVLE‐based nutraceuticals and biomaterials, supporting its potential as an adjuvant in bone tissue engineering and regenerative medicine.

## 4 Conclusion

The present study demonstrates that *Vitis vinifera* leaves extract (VVLE) is a promising natural agent with osteogenic potential to enhance physiological pathways of the human fetal osteoblasts mineralization and differentiation. These findings suggest a substance for the development of dietary supplements derived from *Vitis vinifera* leaves extract for bone health applications, including preventive and supportive strategies in bone‐wasting diseases.

Further in vivo studies are needed to confirm VVLE's efficacy and its potential in preserving bone health and preventing bone loss.

## 5 Experimental Section

### 5.1 Cell Culture

Human fetal osteoblast cell line (hFOB 1.19) (American Type Culture Collection, ATCC, Manassas, VA, USA) was used in this study. hFOB cells were cultured in a 1:1 mixture of Ham's F12 medium–Dulbecco's modified Eagle's medium (D8437, Merk Life Science Srl., Milan, Italy), supplemented with 2.5 mM L‐glutamine (G7513, Merk LifeScience Srl, Milan, Italy) and 10% fetal bovine serum (F7524, FBS, Merk Life Science Srl, Milan, Italy), and incubated in a humidified atmosphere containing 5% CO_2_ at 37°C.

To maintain selective growth, 0.3 mg/mL G418 (4727878001, Merk Life Science Srl, Milan, Italy), an aminoglycoside antibiotic, was added to the culture medium, useful for maintaining hFOB 1.19 cells with the neomycin resistance gene.

The medium was replaced twice a week, and cells were split at about 80% of confluence (Calabrese et al. 2023).

### 5.2 *Vitis vinifera* Leaves Extract

*Vitis vinifera* leaves extract (VVLE) (Case Number 84929‐27‐1) was purchased from FarmaLabor Srl (Canosa di Puglia, Barletta, Italy). The product used in this study complies with the European Pharmacopeia standards and the Safety Regulation 2005/396/EC. The technical specifications and certificate of analysis are reported in Supporting Material. VVLE was dissolved in a 1:1 mixture of Ham's F12 Medium and DMEM to solutions at concentrations of 25, 50, and 100 μg/mL.

### 5.3 Cytotoxicity

The cytotoxicity of VVLE in hFOB 1.19 cells was assessed by 3‐[4,5‐dimethylthiazol‐2‐yl]‐2,5‐diphenyl tetrazolium bromide (MTT) assay. The MTT reduction assay was performed to estimate cell metabolic activity as an indicator of cell health (Mosmann 1983; Rizzo et al. 2017).

The hFOB 1.19 cells were seeded in 96‐well microplates (Thermofisher, Korea) at a density of 1 × 105 cells/well in 1:1 mixture of Ham's F12 Medium. The next day, the medium was changed, and the cells were grown in the presence of various concentrations of VVLE (25–100 μg/mL). After 24 and 48 h of incubation, the cells were washed with PBS without calcium and magnesium, and the respiratory activity was measured by adding 200 μL of MTT solution (1 mg/mL in FBS‐free medium) (M5655, Sigma‐Aldrich, USA). After 2 h incubation at 37°C and 5% CO_2_, the formed crystals were solubilized in 200 μL of DMSO. The absorbance at 540 nm was read using a synergy HT plate reader (BioTek Instruments Inc., VT, USA). The values are expressed as percentages of cell viability compared to the control (cells incubated without VVLE). To monitor cell confluence after 48 h of incubation, 0.5% crystal violet was used. Images were acquired using a Leica DMi1 inverted microscope equipped with a FLEXACAM C1 12 MP stand‐alone camera (Leica Camera AG, Wetzlar, Germany).

### 5.4 Extracellular Matrix Mineralization

#### 5.4.1 Alizarin Red Staining

Alizarin red S stain has been used to detect the presence of calcium deposits in hFOB cells grown with and without VVLE, according to the manufacturer's protocol (Bernar et al. 2023; Qureshi et al. 2014). Briefly, cells were incubated for up to 14 days in the presence of VVLE at concentrations of 25, 50, and 100 μg/mL. Cells incubated without VVLE were used as a control. Every 2 days, the medium was changed and the VVLE concentration was maintained constant. Thus, the cells were washed with PBS and fixed in 4% PFA for 15 min at room temperature. Then, the cells were washed three times with H_2_O, 2% Alizarin Red S stain solution was added, and the plate was incubated for 30 min at room temperature. Finally, the red dye was discarded, and the cells were washed twice with H_2_O to remove excess dye solution and finally observed by digital inverted microscope Evos M 5000 (Invitrogen, Thermo Fisher Scientific).

The calcium deposits were quantified by using the ImageJ software (Li et al. 2023; Rizzo et al. 2023).

The images were converted to gray scale, and the regions where the dye is present were converted into red pixels. All the analyzed images have the same total number of 3,145,728 pixels. The deposit amount was expressed as the percentage of the red pixels with respect to the total pixels (% highlighted area).

For quantification of Alizarin Red S staining, bound dye was extracted by adding 300 μL of 10% acetic acid and incubating with shaking for 30 min at room temperature. Cells were scraped, transferred to 1.5 mL tubes, shaken, heated to 85°C for 10 min, and centrifuged at 20,000 g for 15 min. Supernatants were transferred to new tubes, neutralized with 200 μL of 10% ammonium hydroxide, and absorbance was measured at 405 nm.

Colorimetric analysis was directly integrated with alkaline phosphatase (ALP) enzymatic activity by alkaline phosphatase activity assay in response to VVLE.

#### 5.4.2 Alkaline Phosphatase Activity Assay

Alkaline phosphatase (ALP) enzymatic level was measured using a colorimetric method based on the conversion of p‐nitrophenyl phosphate into p‐nitrophenol using Alkaline Phosphatase Assay Kit (ABCAM ab83369). The assay was acquired at 405 nm with an HT plate reader (BioTek Instruments Inc., VT, USA) and expressed as ALP level (nmol) obtained by standard curve according to the manufacturer's instructions.

### 5.5 Gene Expression Profile of hFOB Cells

Expression of bone‐related genes was analyzed by quantitative real‐time PCR (qRT‐PCR) at 1, 7, and 14 days. The RNA was isolated from samples using Trizol Reagent (LifeTechnologies, Carlsbad, CA, USA), according to the manufacturer's instructions. The reverse transcription (RT) was carried out in a 20 mL reaction mixture, containing 1× reaction buffer, 0.5 mM dNTP, 20 pmol primers, 3 mM MgCl2, 20 U RNAase inhibitor, and 200 U Improm II reverse transcriptase (Promega Corporation). qRT‐PCR was carried out in a 20 mL reaction mixture containing 1 mL of cDNA preparation, 0.5 mM of each forward and reverse primer, and 10 mL of SsoAdvanced Universal SYBR1 Green Supermix (2×) (Bio‐Rad Laboratories, Hercules, CA, USA). The amplification was carried out using a 7500 Fast Real‐Time PCR System under the following conditions: 95°C for 3 min, followed by 35 cycles of 95°C for 15 s, 55°C for 30 s, and 72°C for 15 s. A melting curve analysis was performed using the instrument's default settings. For this study, gene expression data were analyzed by the 2^−ΔΔCt^ method. The gene expression data were normalized to GAPDH and expressed as the fold ratio relative to the control. Oligonucleotide sequences are reported in Table 1 (Anderson et al. 2011; Ariffin et al. 2022; Rizzo et al. 2023; Safari, Aghanejad, et al. 2022; Shao et al. 2018).

**TABLE 1:** Primer sequence (5′–3′) used in qRT‐PCR.

Protein name	Target gene	Forward	Reverse
Glyceraldehyde 3‐phosphate dehydrogenase	GAPDH	AACAGCGACACCCACTCCTC	CATACCAGGAAATGAGCTTGACAA
Alkaline phosphatase	ALP	ACCATTCCCACGTCTTCACATTT	AGACATTCTCTCGTTCACCGCC
Osteocalcin	β‐GLAP	CAAAGGTGCAGCCTTTGTGTC	TCACAGTCCGGATTGAGCTCA
Osteopontin	SPP1	GCAGACCTGACATCCAGTACC	GATGGCCTTGTATGCACCATTC
Bone sialoprotein	BSP	GGCAGTAGTGACTCATCCGAAG	GAAAGTGTGGTATTCTCAGCCTC
Bone morphogenetic protein 2	BMP2	ACTCGAAATTCCCCGTGACC	CCACTTCCACCACGAATCCA
Extracellular signal‐regulated kinases	ERK	GAACTCCAAGGGCTATACCAAGT	GGAGGGCAGAGACTGTAG GTAGT

### 5.6 Western Blots

hFOB cell lysates were used to investigate the following primary antibodies: anti‐alkaline phosphatase (Thermo Fisher, M0806‐10), anti‐osteocalcin (Merck‐Millipore, AB10911), anti‐osteopontin (Abcam EPR21139‐316), anti‐bone sialoprotein (Thermo Fisher PA5‐114915), anti‐bone morphogenetic protein 2 (Abcam ab214821), anti‐extracellular signal‐regulated kinases (R&D System MAB1576), in 1× PBS, 5% w/v nonfat dried milk, and 0.1% Tween‐20 at 4°C overnight. As directed by the manufacturer, signals were evaluated using an enhanced chemiluminescence (ECL) detection system reagent (Thermo, Monza, Italy). Using BIORAD ChemiDoc TM XRS+ software and densitometry, the relative expression of the protein bands was measured and standardized to the levels of glyceraldehyde 3‐phosphate dehydrogenase (GAPDH).

### 5.7 Statistical Analysis

The data were derived from three independent experiments conducted in triplicate. All the data are expressed as the means ± standard deviation. Data were analyzed using one or two‐way ANOVA followed by Bonferroni's correction unless otherwise stated. These analyses were performed using GraphPad Prism 8. A *p* value of 0.05 or less was regarded as significant.

## Author Contributions

**Maria Giovanna Rizzo:** conceptualization (lead), data curation (equal), formal analysis (equal), writing – original draft (equal). **Marika Cordaro:** project administration (equal), supervision (equal), writing – review and editing (equal). **Dario Morganti:** methodology (equal), software (equal). **Enrico Gugliandolo:** investigation (equal). **Roberta Fusco:** investigation (equal). **Ylenia Marino:** investigation (equal). **Gianluca A. Franco:** formal analysis (equal). **Salvatore Cuzzocrea:** funding acquisition (equal), resources (equal). **Rosanna Di Paola:** project administration (equal), supervision (equal), writing – review and editing (equal). **Sabrina Conoci:** funding acquisition (equal), resources (equal).

## Conflicts of Interest

The authors declare no conflicts of interest.

## Data Availability

Data will be made available on request.
